# Influenza‐like illness in individuals treated with immunosuppressants, biologics, and/or systemic corticosteroids for autoimmune or chronic inflammatory disease: A crowdsourced cohort study, France, 2017–2018

**DOI:** 10.1111/irv.13148

**Published:** 2023-06-28

**Authors:** Ségolène Greffe, Caroline Guerrisi, Cécile Souty, Ana‐Maria Vilcu, Gilles Hayem, Félicie Costantino, Ilaria Padovano, Isabelle Bourgault, Salim Trad, Matthieu Ponsoye, Eve Vilaine, Marion Debin, Clément Turbelin, Thierry Blanchon, Thomas Hanslik

**Affiliations:** ^1^ Department of Internal Medicine Ambroise‐Paré Hospital, Assistance Publique‐Hôpitaux de Paris (AP‐HP) Boulogne‐Billancourt France; ^2^ Sorbonne Université, INSERM, Institut Pierre Louis d'Épidémiologie et de Santé Publique (IPLESP) Paris France; ^3^ Department of Rheumatology Ambroise‐Paré Hospital, Assistance Publique‐Hôpitaux de Paris (AP‐HP) Boulogne‐Billancourt France; ^4^ Department of Rheumatology Saint‐Joseph Hospital Paris France; ^5^ “Simone Veil – Santé” Medical School, Université de Versailles Saint‐Quentin‐en‐Yvelines Université Paris Saclay Montigny‐le‐Bretonneux France; ^6^ Université Paris‐Saclay, UVSQ, Inserm U1173, Infection et inflammation, Laboratory of Excellence INFLAMEX Montigny‐Le‐Bretonneux France; ^7^ Department of Dermatology Ambroise‐Paré Hospital, Assistance Publique‐Hôpitaux de Paris (AP‐HP) Boulogne‐Billancourt France; ^8^ Department of Internal Medicine Foch Hospital Suresnes France; ^9^ Department of Nephrology Ambroise‐Paré Hospital, Assistance Publique‐Hôpitaux de Paris (AP‐HP) Boulogne‐Billancourt France

**Keywords:** crowdsourcing, epidemiological monitoring, immunocompromised patients, influenza incidence, influenza vaccine

## Abstract

**Background:**

Influenza‐like illness (ILI) incidence estimates in individuals treated with immunosuppressants and/or biologics and/or corticosteroid for an autoimmune or chronic inflammatory disease are scarce. We compared the ILI incidence among immunocompromised population and the general population.

**Method:**

We conducted a prospective cohort study during the 2017–2018 seasonal influenza epidemic, on the GrippeNet.fr electronic platform, which allows the collection of epidemiological crowdsourced data on ILI, directly from the French general population. The immunocompromised population were adults treated with systemic corticosteroids, immunosuppressants, and/or biologics for an autoimmune or chronic inflammatory disease, recruited directly on GrippeNet.fr and also among patients of the departments of a single university hospital that were asked to incorporate GrippeNet.fr. The general population consisted of adults reporting none of the above treatments or diseases participating in GrippeNet.fr. The incidence of ILI was estimated on a weekly basis and compared between the immunocompromised population and the general population, during the seasonal influenza epidemic.

**Results:**

Among the 318 immunocompromised patients assessed for eligibility, 177 were included. During the 2017–2018 seasonal influenza epidemic period, immunocompromised population had 1.59 (95% CI: 1.13–2.20) higher odds to experience an ILI episode, compared to the general population (*N* = 5358). An influenza vaccination was reported by 58% of the immunocompromised population, compared to 41% of the general population (*p* < 0.001).

**Conclusion:**

During a seasonal influenza epidemic period, the incidence of influenza‐like illness was higher in patients treated with immunosuppressants, biologics, and/or corticosteroids for an autoimmune or chronic inflammatory disease, compared to the general population.

## 1 INTRODUCTION

Individuals treated with systemic corticosteroids and/or immunosuppressants and/or biologics for an autoimmune or chronic inflammatory disease are at higher risk for developing a severe form of influenza, justifying their annual vaccination against seasonal influenza.^1^, ^2^, ^3^, ^4^, ^5^ Therefore, in France and in many countries, guidelines recommend influenza vaccination for immunocompromised patients.^6^, ^7^, ^8^, ^9^ Despite these recommendations, the influenza vaccination coverage is insufficient in this population.^10^, ^11^

Although influenza is potentially more severe in immunocompromised individuals, the increase in the risk of occurrence of influenza and influenza‐like illness (ILI) in immunocompromised people, compared to the general population, remains poorly estimated and documented. Individuals with certain autoimmune or chronic inflammatory diseases appeared to have an increased risk of contracting influenza. This was specifically described in a large administrative retrospective study conducted in the United States in a cohort of patients with rheumatoid arthritis and in a study conducted in Sweden in a cohort of patients with ANCA‐associated vasculitis, where a significant increase in the incidence of influenza compared to the general population has been shown.^3^, ^12^ However, to our knowledge, influenza or ILI incidence in a global population of immunocompromised individuals with autoimmune or chronic inflammatory diseases treated with immunosuppressants, biological agents, and/or systemic corticosteroids has not been compared to the general population.

Traditionally, influenza surveillance is based on clinical and virological surveillance networks, such as sentinel networks. This type of surveillance is not tailored to permit a specific focus on the targeted groups for which the influenza vaccination is recommended. Since 2012, a population‐based surveillance system, named GrippeNet.fr, has supplemented the traditional influenza surveillance systems by providing ILI information directly from the general population, through the Internet.^13^, ^14^ This system already allowed the specific follow‐up of individuals targeted by the influenza vaccination recommendations, such as pregnant women.^14^, ^15^

In this study, thanks to the GrippeNet.fr platform, a specific monitoring of individuals treated with systemic corticosteroids and/or immunosuppressants and/or biologics for an autoimmune or chronic inflammatory disease was implemented during the 2017–2018 influenza season, with the main objective to estimate the ILI incidence during the 2017–2018 seasonal influenza epidemic, in those immunocompromised individuals compared to the general population (i.e., patients not treated with systemic corticosteroids and/or immunosuppressants and/or biologics for an autoimmune or chronic inflammatory disease). We also estimated the influenza vaccination coverage in both populations.

## 2 METHODS

We conducted a crowdsourced cohort study, based on the use of GrippeNet.fr (https://www.grippenet.fr/), a French online influenza surveillance system integrated into a larger European platform (called InfluenzaNet), running since 2012.^16^ GrippeNet.fr participants are volunteers recruited from the general French population through communication actions (Internet, radio, television, etc.). Individuals register directly on the GrippeNet.fr website with an e‐mail address. This electronic platform allows the collection of epidemiological data on ILI directly from the population each winter period. This study was conducted during the 2017–2018 GrippeNet.fr season, spanning from November 22, 2017, to April 29, 2018.

### 2.1 Immunocompromised population cohort

The immunocompromised population consisted of individuals aged 18 years and over, treated with systemic corticosteroids (≥10 mg/day prednisone equivalent without specification of treatment time duration), immunosuppressants, and/or biologic agents for an autoimmune or chronic inflammatory disease, who agreed to participate. Individuals were required to consent to participate, to have an active e‐mail address and Internet access.

They were recruited by two modalities:

1. Physicians in various health departments (dermatology, rheumatology, nephrology, and internal medicine) of a university hospital (Ambroise‐Paré Hospital, Boulogne‐Billancourt, France) identified patients they treated with corticosteroids, immunosuppressants, and/or biologics for an autoimmune or chronic inflammatory disease. They sent them a letter specifying the objectives and protocol of the study and invited them to participate anonymously by connecting to the GrippeNet.fr website.
2. A communication campaign was also conducted among all participants of the GrippeNet.fr platform, explaining the objectives and protocol of the study.

To identify immunocompromised individuals, all participants connecting to the GrippeNet.fr website were asked to answer the following specific question: “Are you monitored in a hospital department for an autoimmune or chronic inflammatory disease?” (yes/no). If the answer was “Yes,” additional questions were asked to document their disease and treatments, based on which they were included in the immunocompromised patient's cohort. For confidentiality obligations, we could not distinguish patients recruited by the physician's letter from those recruited directly among the GrippeNet.fr participants.

### 2.2 General population cohort

The general population consisted of individuals aged 18 years and over, participating in GrippeNet.fr and reporting no autoimmune or chronic inflammatory disease nor the use of medications such as corticosteroids or immunosuppressants.

### 2.3 Data collection

After registration on the GrippeNet.fr platform, an inclusion questionnaire was sent to the participants, documenting the following parameters: age, gender, height and weight, level of education, living area, household composition, comorbidities, and influenza vaccination status (according to the national French recommendations, an influenza vaccination voucher, allowing free vaccination, is sent to individuals with at least one of the following characteristics: age ≥65 years, some chronic underlying diseases including autoimmune or chronic inflammatory diseases on immunosuppressive therapy).

Once the inclusion survey was filled, a reminder e‐mail was sent each week to all participants, from November 2017 to April 2018, asking them to complete a weekly questionnaire and informing them on influenza trends throughout the season. In the weekly questionnaire, participants were asked to report any symptoms experienced in the last 7 days. To be included in the study, each participant had to answer to at least one weekly questionnaire.

### 2.4 Data analyses

Demographic and clinical characteristics were described in both the immunocompromised and the general populations. The two cohorts were compared using the Fisher test or the chi‐squared test, with an alpha risk of 5%.

A calculation power was computed in order to determine the minimum number of individuals needed to detect a significant difference of 5%, using a one‐sided test. Since about 28% of GrippeNet.fr participants reports an ILI episode each season,^17^ it would be necessary to include at least 52 immunocompromised patients, if we assume that they are twice as likely to have an ILI compared to the general population.

### 2.5 ILI incidence estimation

The ECDC ILI case definition (European Centre for Disease Prevention and Control) was used, that is, the combination of three criteria: (i) sudden onset of symptoms, accompanied with (ii) at least one systemic sign (among fever or feverishness, headache, myalgia, or asthenia) and (iii) at least one respiratory symptoms (among cough, sore throat, and shortness of breath).

ILI incidence was estimated each week among the two populations (immunocompromised population and GrippeNet.fr participants). The incidence computation method was adapted from the method described in Guerrisi et al.^18^ Incidence time series were adjusted by age groups only (18–44, 45–64, and ≥65 years) to account for the non‐representative nature of the GrippeNet.fr population.^13^ To reduce the participation bias, the first weekly questionnaire of each participant was excluded from the analyses. Indeed, we have previously shown that the first weekly questionnaire completed by a participant most often includes symptoms and that can impact incidence estimations.^18^ In participants reporting an ILI episode in more than one weekly questionnaire during the study period, the episodes were considered different if they were separated by more than 15 days. However, participants were still able to have more than one ILI episode during the season. The weekly incidence rate was computed as the number of reported ILI episodes divided by the total number of participants for a given week. Estimates of incidence were calculated for the immunocompromised population and for the general population. Incidence comparisons between immunocompromised population and the general population were computed using the chi‐squared test.

Data from the French *Sentinelles* network (www.sentiweb.fr) were used to identify the 2017–2018 influenza seasonal epidemic period.^19^ The *Sentinelles* network conducts primary care ILI surveillance, thanks to data collected from GPs consultations. It is a referral network for the detection of winter influenza epidemics and has been previously used as a validation tool for GrippeNet.fr incidence estimations.^18^, ^19^

### 2.6 Ethical approval

GrippeNet.fr was reviewed and approved by the French Advisory Committee on the Processing of Information in Health Research (i.e., CCTIRS, authorization 11.565) and by the French National Commission on Informatics and Liberties (CNIL, authorization DR‐2012‐024). Participant consent is informed and provided through registration.

## 3 RESULTS

**FIGURE 1:**
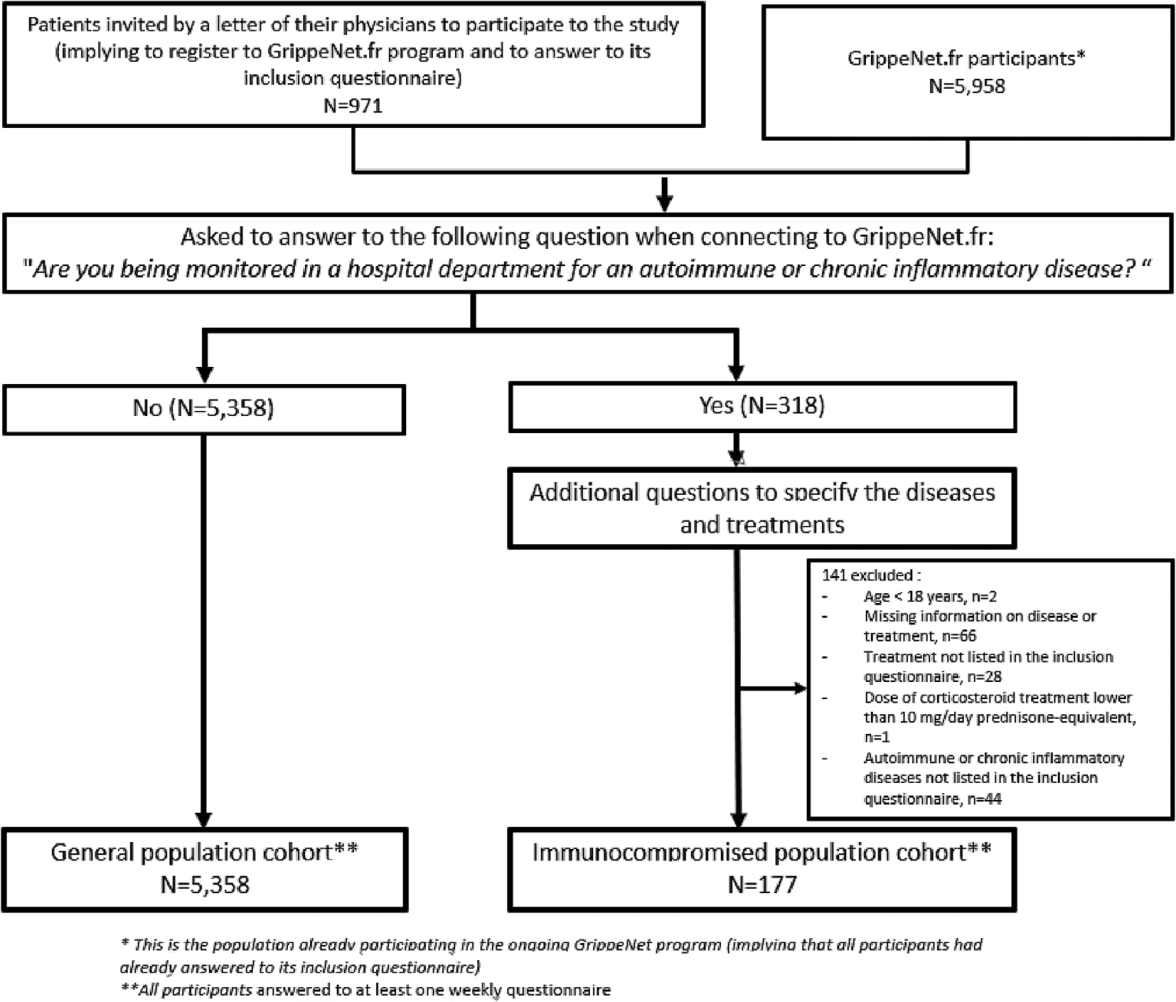
Flowchart of the study.

After screening, 318 patients reported being monitored in a hospital department for an autoimmune or chronic inflammatory disease, of which 177 were included because they met the inclusion criteria (Figure 1). The general population cohort included 5358 individuals (Figure 1). Thereafter, the immunocompromised and GrippeNe.fr participants completed a total number of 2324 and 85 301 weekly questionnaires, respectively (i.e., a median number of 15 [interquartile range (IQR): 5–20] and 19 [IQR: 11–22] weekly questionnaires, respectively).

The immunocompromised individuals were treated mainly for rheumatoid arthritis (*n* = 54, 31%), spondyloarthritis (*n* = 38, 21%), and psoriasis arthritis (*n* = 29, 16%) (Table 2). They received either a monotherapy in 111 cases (biologic [*n* = 52, 29%], immunosuppressant [*n* = 31, 18%], systemic corticosteroids at more than 10 mg/day of prednisolone‐equivalent [*n* = 28, 16%]) or a combination of treatments in 66 cases (37%) patients (Table 1). Individuals who received monotherapy treatment were 32% (36/111) to experience at least one ILI, while 36% (24/66) of individuals receiving a combined therapy had at least one ILI. This difference was not significant (*p* = 0.62).

**TABLE 1:** Diseases and treatments of the immunocompromised population.

	Immunocompromised population (*n* = 177)
Diseases being treated with systemic corticosteroids and/or immunosuppressants and/or biologic agents	177
Rheumatoid arthritis	54 (31%)
Spondyloarthritis	38 (21%)
Psoriasis arthritis	29 (16%)
Giant cell arteritis	7 (4%)
Granulomatosis with polyangiitis	5 (3%)
Sjögren's syndrome	4 (2%)
SAPHO syndrome (synovitis‐acne‐pustulosis‐hyperostosis‐osteitis syndrome)	5 (3%)
Others^a^	35 (20%)
Treatments	
Monotherapy	111 (63%)
Biologic agent^b^	52 (29%)
Immunosuppressant agent^c^	31 (18%)
Corticosteroids monotherapy^d^	28 (16%)
Combination therapy	66 (37%)
Corticosteroids + Biologic agent	13 (7%)
Corticosteroids + Immunosuppressant agent	21 (12%)
Immunosuppressant agent + Biologic agent	19 (11%)
Corticosteroids + Immunosuppressant agent + Biologic agent	13 (7%)

^a^ Others: Multiple sclerosis (*n* = 4), lupus (*n* = 2), ulcerative colitis (*n* = 2), autoimmune polyendocrinopathy (*n* = 2), systemic sclerosis (*n* = 1), polymyalgia rheumatica (*n* = 1), hypereosinophilic syndrome (*n* = 1), lichen (*n* = 1), relapsing polychondritis (*n* = 1), alopecia areata (*n* = 1), polyarteritis nodosa (*n* = 1), Behçet disease (*n* = 1), uveitis (*n* = 1), dermatomyositis (*n* = 1), Still's disease (*n* = 1), mixed connective tissue disease (*n* = 1), more than one disease (*n* = 2), and unspecified disease for which the patient is being treated with systemic corticosteroids and/or immunosuppressants and/or biologic agents (*n* = 11).

^b^ TNF inhibitors (infliximab, etanercept, adalimumab, certolizumab pegol, golimumab, and bélimumab) and other biologic agents (rituximab, anakinra, tocilizumab, secukinumab, ustekinumab, and abatacept).

^c^ Methotrexate, cyclophosphamide, azathioprine, mycophenolate mofetil, leflunomide, and teriflunomide.

^d^ ≥10 mg/day of prednisone equivalent.

Immunocompromised individuals had a mean age of 57 ± 13 years, compared to 56 ± 15 years in the general population (*p* = 0.15). Compared to the individuals from the general population, they lived more frequently in urban areas (88% versus 81%, respectively, *p* = 0.02) and were more often smokers (17% versus 11%, respectively, *p* = 0.03), overweight (30% versus 28%, respectively, *p* = 0.03), and obese (16% versus 12%, respectively, *p* = 0.03); no difference in the prevalence of comorbidities was observed (23% versus 22%, *p* = 0.08) (Table 2).

**TABLE 2:** Characteristics of the immunocompromised population and the GrippeNet.fr participants.

	Immunocompromised population (*n* = 177)	GrippeNet.fr participants (*n* = 5358)	*p*‐value
Gender	m.d. = 0	m.d. = 0	
Male	62 (35%)	2026 (38%)	0.50
Female	115 (65%)	3332 (62%)	
Age	m.d. = 0	m.d. = 0	
18–45	33 (19%)	1395 (26%)	0.01
45–65	92 (52%)	2169 (40%)	
65+	52 (29%)	1794 (33%)	
Residential area	m.d. = 0	m.d. = 0	
Rural	21 (12%)	1032 (19%)	0.02
Urban	156 (88%)	4326 (81%)	
Education	m.d. = 2	m.d. = 43	
<High school diploma	41 (23%)	820 (15%)	0.02
High school diploma	26 (15%)	972 (18%)	
>High school diploma	108 (62%)	3523 (66%)	
Household composition	m.d. = 0	m.d. = 18	0.02
Living alone	43(24%)	908 (17%)	
Living with adults only	93(53%)	2851 (53%)	
Living with children	41(23%)	1581 (30%)	
Main activity	m.d. = 18	m.d. = 71	
Retired	60 (38%)	2014 (38%)	0.06
Working	79 (50%)	2760 (52%)	
Student	2 (1%)	168 (3%)	
Sick or parental leave	14 (9%)	214 (4%)	
Unemployed	4 (3%)	135 (3%)	
Smoker	m.d. = 1	m.d. = 9	
Smoker	30 (17%)	613 (11%)	0.03
Non‐smoker	146 (83%)	4736 (89%)	
Respiratory allergy (m.d. = 0)	m.d. = 0	m.d. = 0	0.38
Yes	58 (33%)	1943 (36%)	
No	119 (67%)	3415 (64%)	
BMI	m.d. = 1	m.d. = 40	
Underweight	11 (6%)	193 (4%)	0.03
Normal	84 (48%)	3007 (56%)	
Overweight	52 (30%)	1493 (28%)	
Obese	29 (16%)	625 (12%)	
Chronic treatment for comorbidity (other than ID)	m.d. = 0	m.d. = 0	
Comorbidity	49 (23%)	1175 (22%)	0.08
No comorbidity	128 (77%)	4183 (78%)	
Asthma	14 (8%)	352 (7%)	0.58
Lung disease	14 (8%)	182 (3%)	p < 10^−2^
Kidney disease	8 (5%)	43 (1%)	p < 10^−3^
Cardiovascular disease	21 (12%)	602 (10%)	p = 0.89
Diabetes	11 (6%)	215 (4%)	0.21
Perceived ILI frequency	m.d. = 8	m.d. = 207	
Rarely	140 (83%)	4339 (84%)	0.59
Often	29 (17%)	812 (16%)	
Transport	m.d. = 0	m.d. = 0	
Private	144 (81%)	4514 (84%)	0.30
Public	33 (19%)	844 (16%)	
At‐risk contacts	m.d. = 0	m.d. = 0	
Yes	99 (56%)	2795 (52%)	0.36
No	78 (44%)	2563 (48%)	
Vaccination voucher received	m.d. = 0	m.d. = 0	
Yes	81 (46%)	2104 (39%)	0,12
No	93 (53%)	3192 (60%)	
Do not know	3 (2%)	62 (1%)	
Influenza vaccination 2017–2018 season	m.d. = 0	m.d. = 12	<10^−3^
Yes	103 (58%)	2205 (41%)	
No	74 (42%)	3141 (59%)	
Influenza vaccination 2016–2017 season	m.d. = 3	m.d. = 44	<10^−3^
Yes	101 (57%)	2272 (43%)	
No	73 (41%)	3042 (57%)	

Abbreviation: m.d., missing data.

Regarding influenza vaccination, 46% (*n* = 81) of the immunocompromised individuals reported that they received a voucher from the national social health Insurance system, compared to 39% (*n* = 2104) among the general population (*p* = 0.12). A seasonal 2017–2018 and 2016–2017 influenza vaccination was reported by 58% (*n* = 103) and 57% (*n* = 101) of the immunocompromised individuals respectively, compared to 41% (*n* = 2205) and 43% (*n* = 2272) of the general population, respectively (*p* < 0.001). No ILI rate differences were observed between the immunocompromised population cohort and the GrippeNet.fr participants regarding the vaccination status (*p* = 0.69 and 0.76 for the vaccinated and unvaccinated respectively).

### 3.1 ILI incidence

At least one ILI episode was reported during the entire GrippeNet.fr season by 60 (34%) immunocompromised individuals and 1474 (28%) GrippeNet.fr participants. Among those who had at least one ILI, 37 (62%) immunocompromised individuals and 1045 (71%) GrippeNet.fr participants have had just one ILI over the season.

**FIGURE 2:**
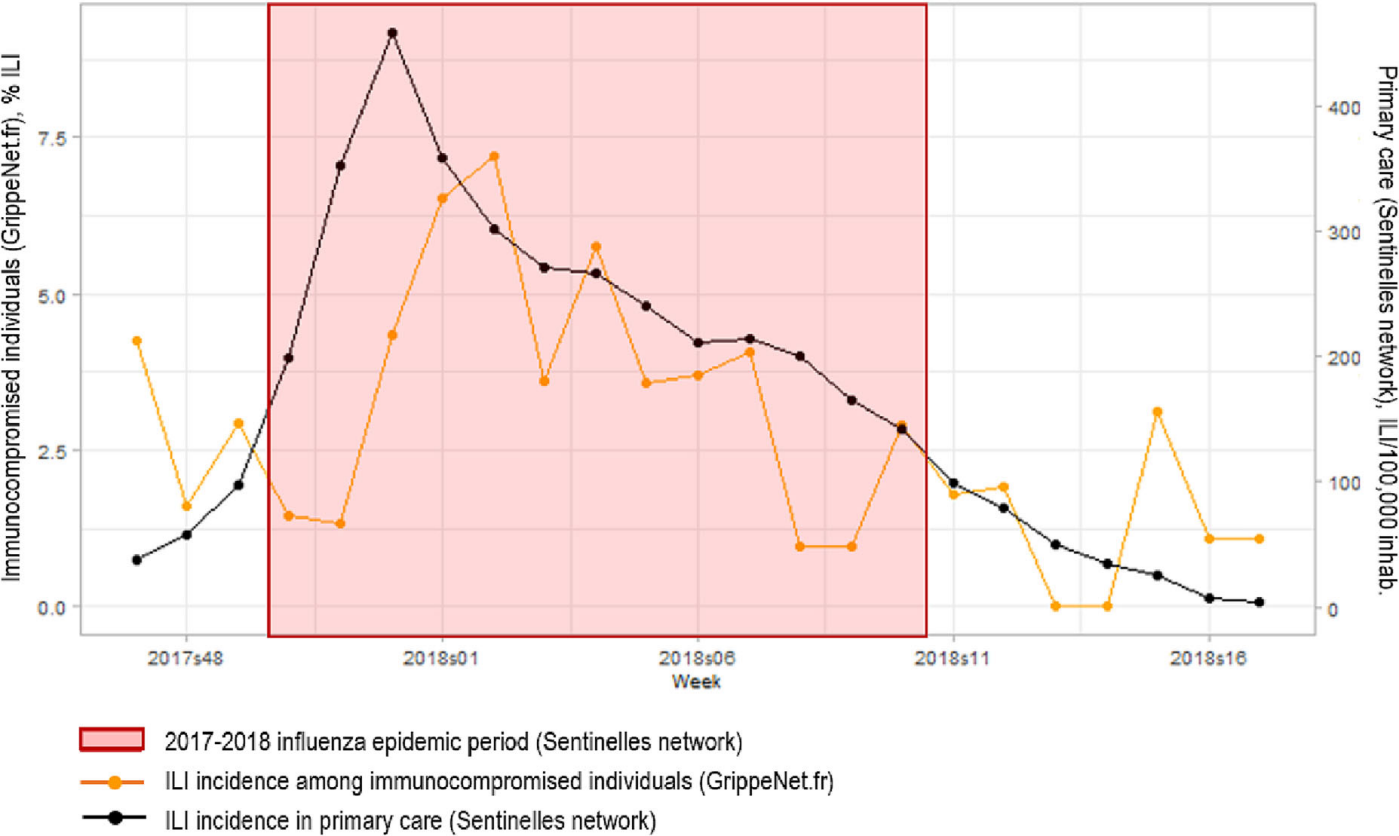
Weekly ILI incidence, in immunocompromised individuals (GrippeNet.fr platform) and in primary care (*Sentinelles* network), 2017–2018 season.

ILI incidence trends in immunocompromised individuals were similar to those observed through the traditional outpatient‐based surveillance of the *Sentinelles* network (Figure 2). The ILI peak was reached on week 2018w02 among the immunocompromised individuals, that is, 2 weeks after the epidemic peak reported by the *Sentinelles* network.

**FIGURE 3:**
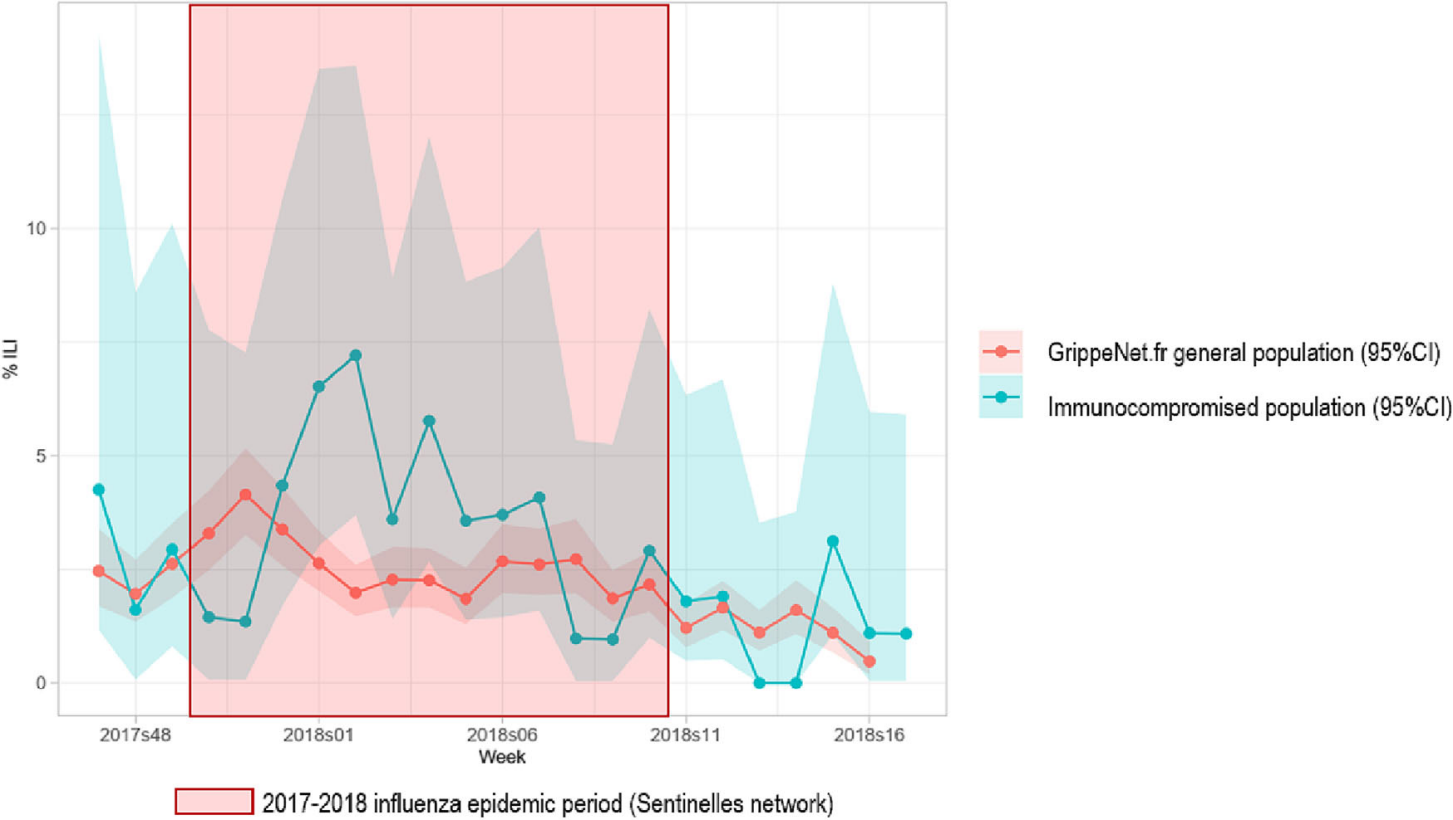
Weekly incidence of ILI episodes reported on the GrippeNet.fr platform, in immunocompromised individuals and the general population, 2017–2018 season.

Figure 3 presents the weekly incidence rates of ILI episodes estimated in both the immunocompromised and the general study populations. During the 2017–2018 seasonal influenza epidemic (weeks 2017w52 to 2018w07), 52 (31%) immunocompromised individuals and 1115 (21%) GrippeNet.fr participants experienced at least one ILI episode (*p* = 0.008). Immunocompromised individuals had 1.59 (95% CI: 1.13–2.20) higher odds to experience an ILI episode during the seasonal influenza epidemic.

## 4 DISCUSSION

During the 2017–2018 seasonal influenza epidemic, ILI was more frequent in individuals treated with systemic corticosteroids and/or immunosuppressants and/or biologic agents for an autoimmune or chronic inflammatory disease, compared to the general population.

To our knowledge, this is the first population‐based study that estimates the incidence of ILI in a prospective cohort of immunocompromised individuals compared to the general population. In this study, immunocompromised individuals were treated mainly for an autoimmune inflammatory rheumatic disease (AIRD). Previous cross‐sectional studies have already shown that individuals with AIRDS were at higher risk for contracting influenza compared to the general population, but did not allow to answer clearly to the question whether the reported differences in incidences were the results of the treatment or of the underlying AIRD.^2^, ^3^, ^4^, ^5^, ^20^, ^21^, ^22^ For example, an administrative study conducted in the United States concluded that rheumatoid arthritis was associated with increased incidence of seasonal influenza and its complication but did not linked this increase to the use of disease‐modifying anti‐rheumatic drugs or biologics.^3^ On the other hand, a Swedish cohort of ANCA‐associated vasculitis showed a significant increase in the incidence of influenza in patients with vasculitis compared to the general population but did not study the impact of medications on the risk of influenza infection.^20^

This higher incidence of ILI (as a proxy of influenza infections) observed among the immunocompromised population cohort could have been due to exposure variations. Although it is not possible to give a clear estimation of exposure based on our study, we assumed the exposure to influenza infections was the same between the immunocompromised and GrippeNet.fr participants. Indeed, the two populations did not differ for various socio‐demographic characteristics (public transportation, employment, and at‐risk contacts), which could have impacted the exposure to influenza infections. Moreover, the immunocompromised reported to live alone more frequently than the GrippeNet.fr participants and that could have been a protective factor. The place of residency could have played a role regarding influenza infections exposure (immunocompromised being more urban than the GrippeNet.fr participants and then at a higher risk of exposure). However, we have shown in a previous work that the residential area was not associated with ILI.^17^ Finally, as the study took place before the COVID‐19 pandemic, there should be no behavioral differences regarding the use of face masks to protect against epidemics because immunocompromised people did not have specific recommendations compared to the general population.

We have also highlighted that immunocompromised individuals were significantly better vaccinated than the general population, although more than half of them declared not having received a vaccination voucher from the French Healthcare system. Our results are similar to previous works focusing on immunocompromised patients, for whom an insufficient influenza vaccine coverage has already been observed.^10^, ^11^ Although it appears that most physicians treating immunocompromised population are aware of the specific targeted recommendations regarding influenza vaccination,^23^ there remains a significant proportion of unvaccinated individuals.

Our study had some limitations. First, there was no influenza virological confirmation among individuals presenting ILI symptoms. However, it has been shown that ILI reported in *Sentinelles* network surveillance were caused by influenza viruses in around 70% of cases during the seasonal epidemics.^19^, ^24^ Thus, it can be assumed that here, most of the ILI cases reported were actually due to influenza infection. Second, our study was limited to the 2017–2018 influenza epidemic. As for any study on influenza, we cannot anticipate what would have been the results on a different year, even though the epidemic pattern is quite reproducible since many years in France.^19^, ^24^ Third, GrippeNet.fr is only based on declarative data concerning symptoms reported by individuals from the general population, but not validated by physicians. Therefore, this could have induced declaration biases. However, we tried to limit this bias by collecting only simple symptoms that are easily identifiable by the participants instead of asking for ILI syndrome. Moreover, it has been previously shown that epidemic trends from the GrippeNet.fr population were correlated with those from the French *Sentinelles* network.^18^ In this context, GrippeNet.fr and other similar crowdsourced platforms have been used to follow influenza epidemics using the ILI indicator as influenza virus circulation proxy.^16^, ^18^, ^25^, ^26^ Fourth, the small sample size of immunocompromised patients could also have limited our conclusions. Fifth, the fact that the immunocompromised people participating in the GrippeNet.fr cohort are volunteers could have induced a bias in the representativeness of the overall immunocompromised population in France. The GrippeNet.fr cohort representativeness has been previously studied^13^ showing a higher influenza vaccination coverage compared to the general population. This could have been similar in the immunocompromised population. However, our study helped to have a first point estimate of influenza vaccination coverage in this specific immunocompromised population. Also, the volunteering effect probably did not impact the incidence trends because we adjusted on first‐time reporting bias and on age groups to limit its impact on ILI incidence estimations, as shown in a previous work.^18^ Sixth, the effectiveness of influenza vaccination in both populations could not be assessed here. Seventh, considering that exposure to the influenza virus is the same in both populations, the incidence of ILI in immunocompromised population cohort could be even higher. Indeed, previous works have shown that immunocompromised individuals would be more likely to get asymptomatic flu, but this cannot be measured with our study.^27^ Moreover, we cannot distinguish in our study whether the differences in incidences were the result of the treatment or of the underlying autoimmune disease, as the inclusion criteria were to have both. Finally, this study was not designed to estimate if the higher risk of ILI reported by ambulatory immunocompromised patients translates into higher risks of hospitalization or mortality.

In conclusion, during the 2017–2018 seasonal epidemic in France, the incidence of ILI was higher among individuals treated with immunosuppressants, biologics, and/or systemic corticosteroids for an autoimmune or chronic inflammatory disease compared to individuals that were not treated for an autoimmune or chronic inflammatory disease.

## AUTHOR CONTRIBUTIONS

**Ségolène Greffe**: Conceptualization; investigation; writing—original draft; writing—review and editing; formal analysis. **Caroline Guerrisi**: Data curation; formal analysis; methodology; writing—review and editing. **Cécile Souty**: Formal analysis; writing—review and editing. **Ana‐Maria Vilcu**: Formal analysis; writing—review and editing. **Gilles Hayem**: Data curation; writing—review and editing. **Félicie Costantino**: Data curation; writing—review and editing. **Ilaria Padovano**: Data curation; writing—review and editing. **Isabelle Bourgault**: Data curation; writing—review and editing. **Salim Trad:** Data curation; writing—review and editing. **Matthieu Ponsoye:** Data curation; writing—review and editing. **Eve Vilaine**: Data curation; writing—review and editing. **Marion Debin**: Formal analysis; methodology; writing—review and editing. **Clément Turbelin**: Formal analysis; writing—review and editing. **Thierry Blanchon**: Writing—review and editing. **Thomas Hanslik**: Writing—original draft; writing—review and editing.

## CONFLICT OF INTEREST STATEMENT

No financial or non‐financial benefits have been received or will be received from any party directly or indirectly related to this article's subject.

### PEER REVIEW

The peer review history for this article is available at https://www.webofscience.com/api/gateway/wos/peer-review/10.1111/irv.13148.

## Data Availability

Data will be made available on reasonable request.
